# Eu_2_O_3_@Cr_2_O_3_ Nanoparticles-Modified Carbon Paste Electrode for Efficient Electrochemical Sensing of Neurotransmitters Precursor L-DOPA

**DOI:** 10.3390/bios13020201

**Published:** 2023-01-29

**Authors:** Aleksandar Mijajlović, Miloš Ognjanović, Dragan Manojlović, Filip Vlahović, Slađana Đurđić, Vesna Stanković, Dalibor Stanković

**Affiliations:** 1 Faculty of Chemistry, University of Belgrade, Studentski trg 12-16, 11000 Belgrade, Serbia; 2 “VINČA” Institute of Nuclear Sciences—National Institute of the Republic of Serbia, University of Belgrade, 11000 Belgrade, Serbia; 3 Center for Nanotechnologies, South Ural State University, Lenin Prospekt 76, 454080 Chelyabinsk, Russia; 4 Institute of Chemistry, Technology and Metallurgy—National Institute of the Republic of Serbia, University of Belgrade, Njegoševa 12, 11000 Belgrade, Serbia

**Keywords:** levodopa, electrochemical sensor, rare earth nanomaterial, hydrothermal synthesis, micromolar detection

## Abstract

There are ten million people in the world who have Parkinson’s disease. The most potent medicine for Parkinson’s disease is levodopa (L-DOPA). However, long-term consumption of L-DOPA leads to the appearance of side effects, as a result of which the control and monitoring of its concentrations are of great importance. In this work, we have designed a new electrochemical sensor for detecting L-DOPA using a carbon paste electrode (CPE) modified with Eu_2_O_3_@Cr_2_O_3_ composite nanoparticles. Rare earth elements, including Eu, are increasingly used to design new electrode nanocomposites with enhanced electrocatalytic properties. Europium has been considered a significant lanthanide element with greater redox reaction behavior. We conducted a hydrothermal synthesis of Eu_2_O_3_@Cr_2_O_3_ and, for the first time, the acquired nanoparticles were used to modify CPE. The proposed Eu_2_O_3_@Cr_2_O_3_/CPE electrode was investigated in terms of its electrocatalytic properties and then used to develop an analytical method for detecting and quantifying L-DOPA. The proposed sensor offers a wide linear range (1–100 µM), high sensitivity (1.38 µA µM^−1^ cm^−2^) and a low detection limit (0.72 µM). The practical application of the proposed sensor was investigated by analyzing commercially available pharmaceutical tablets of L-DOPA. The corresponding results indicate the excellent potential of the Eu_2_O_3_@Cr_2_O_3_/CPE sensor for application in real-time L-DOPA detection.

## 1. Introduction

Parkinson’s disease is a neurodegenerative disorder that occurs due to the accelerated degeneration and death of nerve cells in the brain, primarily those cells responsible for the production of the neurochemical transmitter dopamine. Dopamine is a neurotransmitter and hormone that carries chemical messages between the brain’s nerve cells, as well as between the brain’s nerve cells and the rest of the body. It is essential for many bodily functions, including memory, motivation, learning, reward, and movement. Due to the reduced potential of nerve cells to produce dopamine and, consequently, its insufficiency in the body, symptoms of Parkinson’s disease appear. Tremor, slowness of movement, limb stiffness (rigidity), and problems with balance are common symptoms that arise as a direct result of insufficient dopamine levels in the human body. However, these symptoms, typical for Parkinson’s disease, usually appear slowly, and as the disease progresses, non-motor symptoms (insomnia, depression, apathy, anxiety, etc.) become more common.

By 2015, Parkinson’s disease had affected 6.2 million people and resulted in about 117,400 deaths globally [1,2]. According to the Parkinson’s Foundation, it is believed that more than ten million people in the world suffer from this disease. Although there is no known cure for this disease, there are medications that are successfully used to reduce the effects of the symptoms [3]. The most potent medicine for Parkinson’s disease is levodopa (L-DOPA). L-DOPA can cross the protective blood–brain barrier and, as a dopamine precursor, is used to provide an alternative source of this compound in the human brain. It is also a common use of this drug in the clinical treatment of symptoms similar to Parkinson’s disease that may develop after encephalitis (brain swelling) or specific nervous system injuries (caused by carbon monoxide or manganese poisoning) [4]. However, recent research indicates some severe side effects manifested due to the long-term consumption of L-DOPA, such as nausea, dyskinesia, paranoia, and schizophrenia [5,6]. For all these reasons, rapid, accurate, and precise detection and quantification of L-DOPA are of great importance. It is important to note that determining the content of L-DOPA in pharmaceutical formulas is also necessary, considering that, due to the presence of moisture and atmospheric oxygen, this compound quickly oxidizes, which leads to the loss of the drug and potency reduction [7].

Various analytical techniques, such as high-performance liquid chromatography (HPLC) [8,9], mass spectrometry [10,11], chemiluminescence [12], spectrophotometry [13,14,15] as well as electrochemical methods [16], have been employed for the monitoring of L-DOPA. Some reported methods (HPLC or MS) possess very high sensitivity and selectivity. However, they also exhibit disadvantages for drug detection due to narrow detection ranges, the need for expertise, and multistep laborious and uneconomical sample preparation procedures, including dialysis, purification, and sample fractionation. Additionally, the abovementioned techniques require very expensive instrumentation. Therefore, it is necessary to develop new analytical approaches for detecting drugs that would have a greater possibility of commercial acceptance, such as electrochemical methods [17].

Analytical approaches based on electrochemistry offer simple, rapid, and, most importantly, reliable and accurate methods suitable for detecting and quantifying various biologically active compounds [18,19,20,21,22]. Electrochemical sensors and biosensors represent powerful analytical tools due to their portability, self-contained nature, and low cost [23]. Carbon paste electrodes (CPE) represent one of the most common working electrodes used in electrochemical research. Carbon, with its properties such as insolubility in water, a very high melting point, and good electrical conductivity, makes it an economical and practical choice for electrode construction [24]. The most significant advantages of CPE are the simplicity of preparation, the reproducible surface area, and a low residual current in wide potential windows [25]. Although these electrodes have several disadvantages (the slower speed of electron transfer, less stability when working at a wide range of potentials, and lower sensitivity and reproducibility), suitable carbon paste modification and functionalization is a typical way to overcome these drawbacks [26]. It is important to emphasize that a simple (rational) modification of the conventional CPE leads to the construction of quantitatively new sensors with changed (desired) properties [27]. Volume-modified CPE maintains the great benefit of using a renewable electrode surface for each measurement, thus enabling the reduction of the determination error due to the adsorption of the analyte on the electrode surface.

Rare earth elements, due to their unique properties of 4f–5d electronic orbitals, have attracted the attention of scientists who are considering the development of nanocomposites based on these elements for electrode modification, which would have the same function as noble metal nanocomposites [28,29]. Certain REEs are already used for electrochemical research [30,31]. Europium has been considered a significant lanthanide element with greater redox reaction behavior through its variable oxidation states [32].

In this work, a composite made of Eu_2_O_3_ and Cr_2_O_3_ nanoparticles was used for the first time to develop and optimize an electrochemical sensor. In order to acquire nanoparticles of this compound, we utilized a hydrothermal method, a typical solution-based approach, as an effective and easy synthetic strategy for creating diverse hierarchical designs of nanoparticles. To the best of our knowledge, this is the first time Eu_2_O_3_@Cr_2_O_3_ nanoparticles have been engaged for electrode modification. The obtained electrode was fully characterized and further used to develop the L-DOPA detection method. The developed sensor demonstrates a dynamic linear range, high sensitivity, and a low-level detection limit toward the target analyte.

## 2. Materials and Methods

### 2.1. Chemicals and Instrumentation

Europium (III) oxide (Eu_2_O_3_), chromium (III) nitrate nonahydrate (Cr(NO_3_)_3_ × 9 H_2_O), sodium hydroxide (NaOH), potassium ferrocyanide (K_4_[Fe(CN)_6_]), potassium ferricyanide (K_3_[Fe(CN)_6_]), potassium chloride (KCl), and L-DOPA were obtained from Sigma-Aldrich and directly used for the experimental investigations without purification. Absolute ethanol (CH_3_CH_2_OH) was used as the medium for hydrothermal synthesis. As a supporting electrolyte, Britton–Robinson buffer solution (BRBS) was prepared by mixing equimolar amounts of phosphoric, acetic, and boric acids (40 mM). NaOH solution (0.2 M) was used for tuning and obtaining the desired pH values of the buffer. A pH meter equipped with a universal glass electrode (Orion 1230, Thermo Fisher Scientific, Waltham, Massachusetts, USA) was used for all pH measurements. For real sample analysis, “Madopar” tablets were obtained from “Belgrade Pharmacy” (Belgrade, Serbia). For the preparation of all solutions, double-distilled water was used.

For electrochemical measurements (cyclic voltammetry, differential pulse voltammetry) the potentiostat/galvanostat Autolab, model PGSTAT302N (Metrohm, the Netherlands) was used. A classical three-electrode system was used with a modified or bare CPE as the working electrode (WE), a platinum plate as a counter electrode (CE), and Ag/AgCl (3 M KCl) as a reference electrode (RE). In addition, electrochemical impedance spectroscopy (EIS) measurements were performed using a potentiostat/galvanostat CHI 760b (CH Instruments, Inc., Austin, Texas, USA).

The morphology and surface properties of Eu_2_O_3_@Cr_2_O_3_ nanoparticles, utilized for electrode modification, were investigated using a field emission-scanning electron microscopy Tescan’s MIRA’s 3rd generation FE-SEM at 20 keV at various magnifications (50.0 kx and 100.0 kx). The samples were prepared by fixation on a holder with conductive tape, vacuum dried, and gold spray-coated using a sputter coater. The average size of Eu_2_O_3_@Cr_2_O_3_ nanoparticles was estimated by measuring the highest internal diameter of individual nanoparticles and their clusters, using the public domain software ImageJ.

The crystal structure of materials was analyzed using X-ray powder diffraction (XRPD) data collected on a high-resolution SmartLab^®^ X-ray diffractometer (manufactured by Rigaku in Japan). The measurements were conducted at an accelerating voltage of 40 kV and a current of 30 mA using CuKα radiation source. The dried powder samples were flattened on a zero-background silicon wafer before the diffraction patterns were collected in the 15–60° 2θ range with a recording speed of 2°/min and a step size of 0.02°.

### 2.2. Hydrothermal Synthesis of Eu_2_O_3_@Cr_2_O_3_

**Scheme 1:**
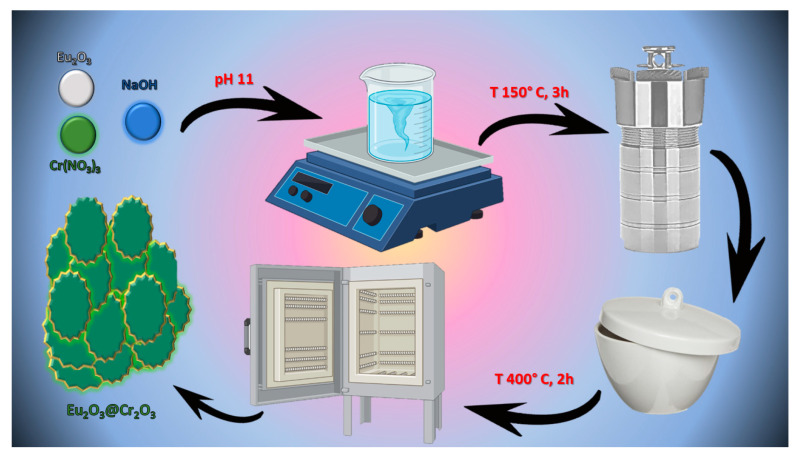
The synthesis of Eu_2_O_3_@Cr_2_O_3_ nanocomposites by the simple hydrothermal-assisted method.

A hydrothermal method was utilized for the production of Eu_2_O_3_@Cr_2_O_3_ nanoparticles. First, the amounts of Eu_2_O_3_ and Cr(NO_3_)_3_ × 9H_2_O needed for synthesis were measured so that the molar ratio of Eu:Cr was equal to 1:2. The corresponding quantity of the mentioned compounds was transferred into a 250-mL beaker. Next, about 30 mL of previously prepared NaOH solution (0.01 M) was added to the beaker so that the pH of the solution was ≤pH = 11. The mixture was stirred for 30 min on a magnetic stirrer and transferred to an autoclave. The obtained material was left in the oven for 3 h at 150 °C. Later, the material was centrifuged (at 5000 rpm, 5 min) and washed with distilled water (3×) and absolute ethanol (3×). The obtained material was transferred to a crucible, heated at 400 °C for 2 h, and then dried in an oven at 80 °C for the next 2 h. After this period, the dried product was collected, ground well manually using the mortar and pestle, and labelled as Eu_2_O_3_@Cr_2_O_3_ nanocomposites. Scheme 1 shows the preparation of Eu_2_O_3_@Cr_2_O_3_ nanocomposite.

### 2.3. Fabrication of Eu_2_O_3_@Cr_2_O_3_/CPE

To improve the electrochemical characteristics of the CPE, its modification was performed by doping it with Eu_2_O_3_@Cr_2_O_3_ nanocomposite. First, the electrode was doped with 10% nanocomposite material by mixing 36 mg of carbon and 4 mg of Eu_2_O_3_@Cr_2_O_3_ nanocomposite. Next, 10 µL of paraffin oil was added to the mixture, and the mixture was homogenized with a pestle for the next 30 min and left for 24 h at room temperature. After 24 h, the electrode was ready for the upcoming electrochemical measurements.

### 2.4. Preparation of the Real Sample

For a real-sample analysis, “Madopar” tablets were well homogenized and accurately weighted, transferred into a 500-mL centrifuge tube, and dissolved in BRBS (pH 2.2). The obtained solution was centrifuged at 5000 rpm for 5 min. Next, the supernatant was diluted with BRBS, and the pH was set to 7. Finally, the supernatant was filtered through a 0.25 μm syringe filter in order to remove suspended particles. Then, the supernatant was taken for further investigation.

## 3. Results

### 3.1. Morphological and Microstructural Characterisation of Eu_2_O_3_@Cr_2_O_3_ Nanocomposite

**Figure 1:**
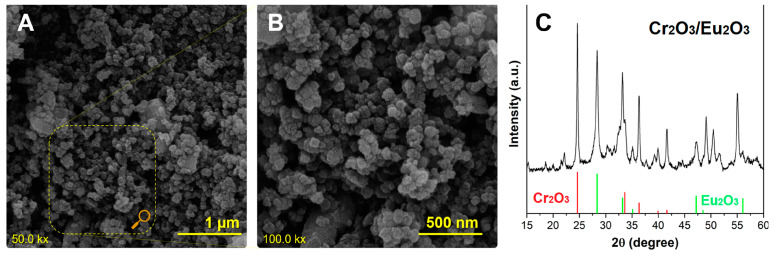
FE-SEM micrographs of cauliflower-like Cr_2_O_3_/Eu_2_O_3_ (**A**) magnification 50 kx, (**B**) 100 kx and (**C**) X-ray diffraction pattern of the prepared nanocomposite. As a reference, the patterns of standard Cr_2_O_3_ (JCPDS #96-210-4123) and Eu_2_O_3_ (JCPDS #96-101-1288) were given below the sample pattern.

FE-SEM micrographs of the material used for electrode modification are shown in Figure 1. The Eu_2_O_3_@Cr_2_O_3_ nanoparticles are grouped into bigger clusters with a strong resemblance to cauliflower (Figure 1B). The average size of nanoflowers is about 150 nm, while individual particles are a couple dozen nanometers in diameter. This specific formation enables them to have a highly specific contact surface, which is a prerequisite for high electrocatalytic activity. The microstructure and phase composition of the materials were checked by XRD (Figure 1C). Most of the reflections match well with the cubic crystal type (space group *I2_1_3*) of europium oxide and the trigonal type (R3-*c* (167) space group) of chromium oxide [33,34]. Therefore, the nanocomposite is composed of a mixture of these two oxides and is hereinafter referred to as Cr_2_O_3_/Eu_2_O_3_.

### 3.2. Electrochemical Characterisation of Eu_2_O_3_@Cr_2_O_3_ Nanocomposite

**Figure 2:**
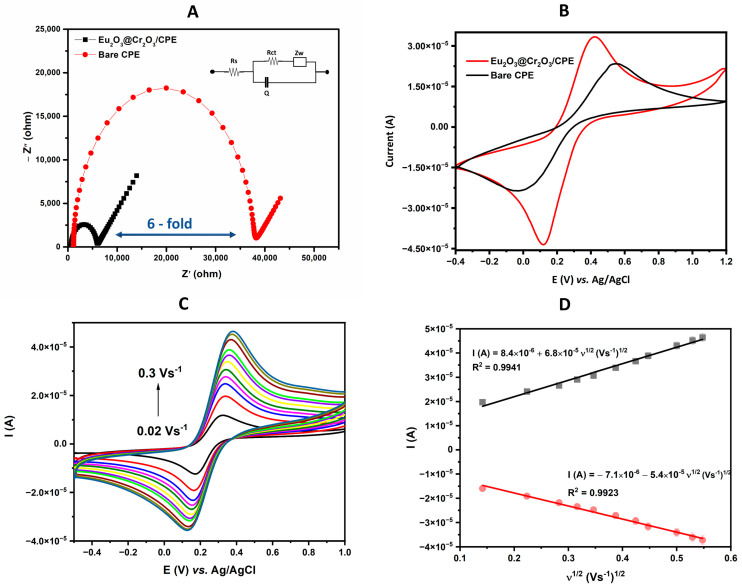
(**A**) EIS curves of bare CPE and Eu_2_O_3_@Cr_2_O_3_/CPE in a 0.1 KCl solution containing 5 mM [Fe(CN)_6_]^3−/4−^ and (**B**) CV curves of bare CPE and Eu_2_O_3_@Cr_2_O_3_/CPE (scan rate 0.05 V s^−1^). (**C**) CV redox current curves for Eu_2_O_3_@Cr_2_O_3_/CPE at scan rates from 0.02 to 0.3 V s^−1^. (**D**) Fitted plots of the redox current response vs. the square root of the scan rates. The aforementioned CV investigations were recorded in 5 mM [Fe(CN)_6_]^3−/4−^ with 0.1 M KCl solution (potential window for CV −0.5 to 1.0 V).

Electrochemical impedance spectroscopy (EIS) is broadly used for assessing a modified electrode’s charge-transfer ability. This method can give information on conductivity/resistance-related properties of the electrode system, such as double-layer capacitance or diffusion rate [35]. EIS measurements were conducted in the 5 mM redox probe containing [Fe(CN)_6_]^3−/4−^ and 0.1 M KCl as a supporting electrolyte, while the frequency range was from 0.01 to 100,000 Hz and the amplitude was 5 mV. Rct values of CPE and Eu_2_O_3_@Cr_2_O_3_/CPE (Figure 2A) were 36,430 Ω and 5124 Ω, respectively. An approximately six-fold decrease in Rct was observed for Eu_2_O_3_@Cr_2_O_3_/CPE, implying an improved material capability to promote interfacial electron transfer. The electrocatalytic activity of Eu_2_O_3_@Cr_2_O_3_ and the extensive surface area of the electrode most likely contribute to the fast electron transfer and enhanced conductivity. Furthermore, the proposed material is characterized by a porous structure and additional catalytic sites, which contribute to better ion diffusion and ion/electron transport, as confirmed by the linear part of the EIS spectra.

Cyclic voltammetry was used to further scrutinize the electron transfer properties and mass transfer ability of bare CPE and Eu_2_O_3_@Cr_2_O_3_/CPE. Figure 2B shows the CV curves recorded with the mentioned electrodes at 0.05 V s^−1^ in 5 mM [Fe(CN)_6_]^3−/4−^ and in 0.1 M KCl as a supporting electrolyte. A pair of well-shaped and symmetrical redox peaks occurred for both electrodes. At bare CPE, a pair of redox peaks (Ipa = 23.50 µA and Ipc = −23.86 µA) can be seen at 0.554 V and −0.026 V, respectively. After modification of the electrode with Eu_2_O_3_@Cr_2_O_3_, peak-to-peak separation was reduced (ΔE was 0.58 V and 0.30 V for bare CPE and Eu_2_O_3_@Cr_2_O_3_/CPE, respectively), while current intensities of redox peaks were increased (Ipa = 33.80 µA and Ipc = −43.52 µA).

The effects of scan rate on the peaks currents were also investigated (Figure 2C) and further used to evaluate the electrochemical surface area of the modified electrode. Both peaks’ current intensities increased with the rise of the scan rate from 0.02 to 0.3 V s^−1^ (Figure 2C). Furthermore, a linear dependence is observed when the current intensity values are plotted as a function of the square root of the scanning rate. The following equations can describe these linear dependences: I (A) = 8.4 × 10^−6^ + 6.8 × 10^−5^ υ^1/2^ (V s^−1^)^1/2^ (R = 0.9941) for Ipa, and I (A) = −7.1 × 10^−6^ − 5.4 × 10^−5^ υ^1/2^ (V s^−1^)^1/2^ (R = 0.9947) for Ipc. These observations indicate the diffusion-controlled nature of the reactions occurring on the Eu_2_O_3_@Cr_2_O_3_-modified CPE. The Randles–Sevick equation, for the reversible electrode process, was employed for the estimation of the electroactive surface of the modified electrode: Ia = 2.69 × 10^5^ × n^2/3^ × A D^1/2^ × ν^1/2^ × C, where Ia is the anodic current peak (A), n stands for the number of electrons transferred in the redox reaction, A is the electroactive area (cm^2^), D is the diffusion coefficient (6.1 × 10^−6^ cm^2^ s^−1^), ν is the scan rate (V s^−1^), and C is the concentration (mM). The calculated surface area for Eu_2_O_3_@Cr_2_O_3_/CPE was 0.029 cm^2^.

### 3.3. Electrochemical Performance of Eu_2_O_3_@Cr_2_O_3_/CPE toward L-DOPA Detection

**Figure 3:**
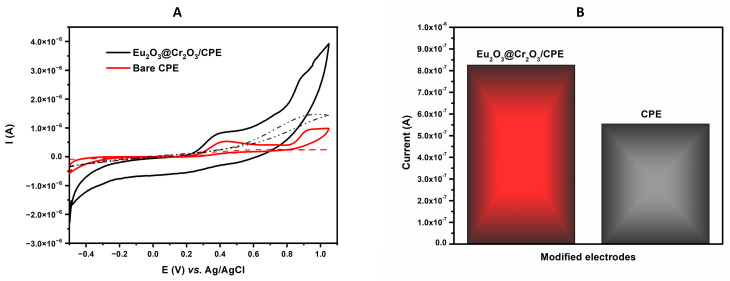
(**A**) CV current response for bare CPE and Eu_2_O_3_@Cr_2_O_3_/CPE, pH 7.0 (BRBS) without (dotted line) and with 100 µM L-DOPA (solid line) at a scan rate of 0.05 V s^−1^. (**B**) The corresponding bar graph for anodic peak current response for L-DOPA over bare and modified CPE.

The application potential of the Eu_2_O_3_@Cr_2_O_3_/CPE for detecting L-DOPA was addressed in the next stage. The electrochemical performances of Eu_2_O_3_@Cr_2_O_3_/CPE and bare CPE toward L-DOPA were investigated in BRBS solution (pH 7.0), containing 100 µM of the analyte, using CV at a scan rate of 0.05 V s^−1^ (Figure 3A). The obtained voltammograms for CPE showed two oxidation peaks, the first at a potential of around +0.40 V and the second at a potential of around +0.90 V. In the case of Eu_2_O_3_@Cr_2_O_3_/CPE, two well-defined oxidation peaks can be noticed at the potential +0.38 V and +0.84 V, respectively, as well as two poorly defined peaks at potential around +0.7 V and +1.0 V. Additional peaks can be assigned to the redox behavior of the chromium presented in the electrode structure [24]. The occurrence of these peaks is an excellent confirmation of the appropriate pathway of electrode preparation and a successful modification procedure. For the first oxidation peak, which has the best oval-shaped structure, the current signal for L-DOPA was significantly higher when the Eu_2_O_3_@Cr_2_O_3_/CPE electrode was utilized (Figure 3B). The decrease in the oxidation potential is a supplementary certificate of the improved interfacial properties of the carbon paste electrode, regarding the effective surface area and diffusion abilities. Thus, all our experiments were focused on this oxidation peak, and the method development for L-DOPA detection was conducted over this peak.

**Figure 4:**
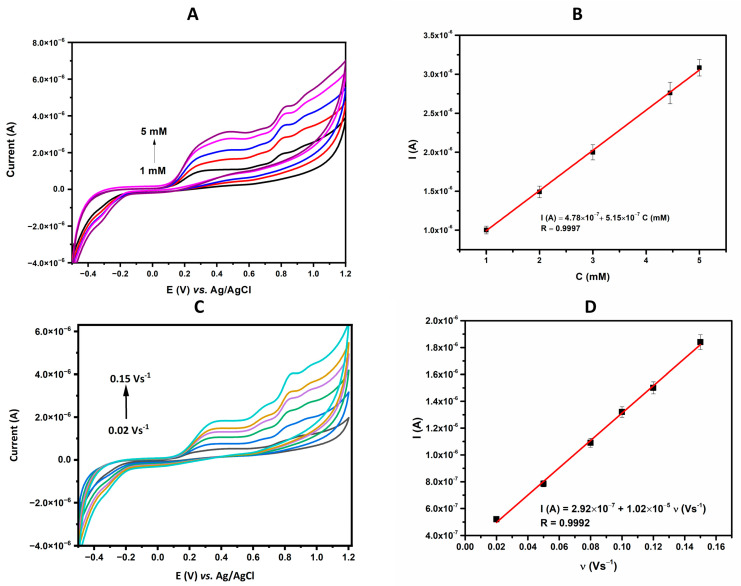
(**A**) CV current response for Eu_2_O_3_@Cr_2_O_3_/CPE at various concentrations of L-DOPA (1 mM–5 mM) at pH = 7.0 (BRBS) at a scan rate of 0.05 V s^−1^. (**B**) The corresponding linear calibration plot for the anodic peak current versus concentrations of L-Dopa. (**C**) CV current response of Eu_2_O_3_@Cr_2_O_3_/CPE at various scan rates (0.02–0.15 V s ^−1^) in BRBS (pH 7.0) with 100 µM of L-Dopa. (**D**) The corresponding plot for redox peak current values against scan rates.

According to the resulting voltammograms (Figure 4A), one can see that the addition of L-DOPA leads to an increase in the oxidation peak current response. The following equation can describe the linear relationship between the oxidation peak current and different concentrations: Ipa (A) = 4.78 × 10^−7^ + 5.15 × 10^−7^ C (mM); R = 0.9997 (Figure 4B). Moreover, we explored the nature of the electrochemical process at the interface electrode/L-DOPA by recording CV using different scan rates (0.02–0.15 V s^−1^) in a solution containing 100 µM L-DOPA in BRBS (pH 7). With an increasing scan rate, the electrode displays a rise in the oxidation peak current (Ipa) of L-DOPA (Figure 4C). In Figure 4D, a linear relationship between current intensities and scan rate was displayed, described by the equation: Ipa (A) = 2.92 × 10^−7^ + 1.02 10^−5^ υ (V s^−1^); R = 0.9992, suggesting that the electrochemical oxidation of L-DOPA is an adsorption-controlled process. Strong adsorption of analytes such as L-DOPA on the carbon electrode surface is expected. However, the proposed sensor developed using volume-modified CPE possesses the great advantage of using a renewable electrode surface after each measurement. Each measurement is made with a new electrode surface, thus minimizing analyte determination errors caused by adsorption.

### 3.4. Effect of Supporting Electrolyte

**Figure 5:**
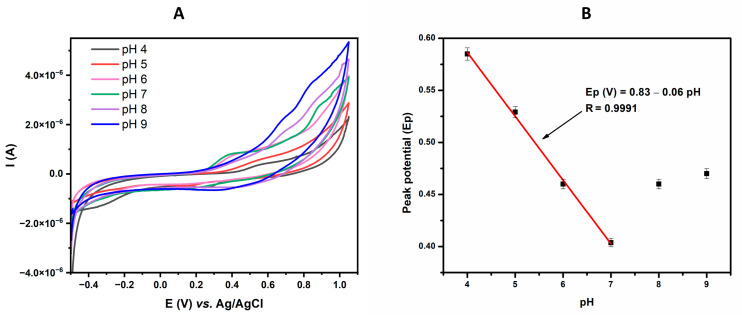
(**A**) CV current responses of Eu_2_O_3_@Cr_2_O_3_/CPE at various pH (4.0–7.0) of BRBS with 100 µM L-DOPA at a scan rate 0.05 V s^−1^. (**B**) The corresponding plot for oxidation peak potentials versus different pH (4.0–7.0).

Effects of different pH values of the supporting solution on the electrocatalytic activity of L-DOPA over Eu_2_O_3_@Cr_2_O_3_/CPE were also investigated. For this purpose, BRBS solutions with different pH values (from 4 to 7) containing 100 µM L-DOPA were used. The acquired voltammograms (Figure 5A) present the changes in peak current intensity and peak potential, depending on the pH value. The peak potential (Epa) shows linearity within the pH range from 4 to 7, followed by the linear regression equation: Epa = 0.8314–0.0613 pH (R = 0.9982). Moreover, the slope value of 61.13 mV/pH is very close to the theoretical Nernstian slope value (−59 mV/pH), indicating that the same number of protons and electrons are involved in the electrochemical oxidation reaction of L-DOPA at Eu_2_O_3_@Cr_2_O_3_/CPE.

### 3.5. Analytical Procedure for L-DOPA Detection

**Figure 6:**
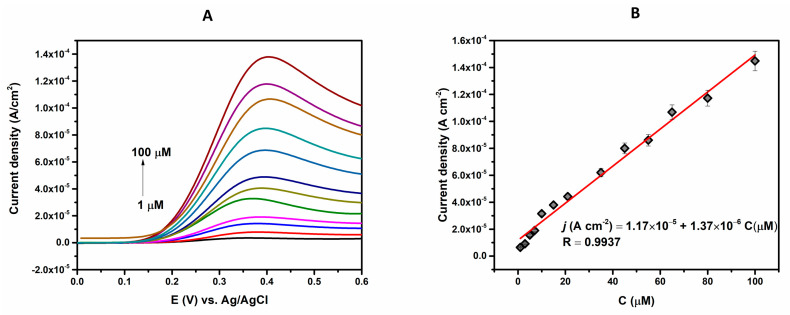
(**A**) DPV oxidation current response for the Eu_2_O_3_@Cr_2_O_3_/CPE towards various concentrations of L-DOPA (1–100 µM) in BRBS (pH 7.0) (**B**) Calibration curve of current response depending on the concentration of L-DOPA in the analyte solution.

Differential pulse voltammetry (DPV) has a considerably higher sensitivity and enhanced resolution than cyclic voltammetry. Consequently, the calibration curve for L-DOPA was obtained using the DPV method. The DPV responses of Eu_2_O_3_@Cr_2_O_3_/CPE for various concentrations of L-DOPA in BRBS at pH 7 are given in Figure 6A. The oxidation peak currents rise linearly with the concentration of the target molecule. The linear relationship between the peak current and L-DOPA concentration is in the range of 1–100 μM, and the following equation describes this connection: I (A) = 3.33 × 10^−7^ + 4.00 × 10^−8^ C (μM) with the correlation coefficient of R = 0.9926 (Figure 6B). The detection limit (LOD) was calculated according to the following equation:

LOD = 3Sb/S

where Sb is the standard deviation of the blank and S is the calibration plot’s slope. The obtained value of LOD was found to be 0.72 µM. The sensitivity value of the proposed sensor was found to be 1.37 µA µM^−1^ cm^−2^ (calculated as a ratio of the calibration slope value and the electroactive surface area of working electrode). All of this implies the good electrocatalytic activity of the Eu_2_O_3_@Cr_2_O_3_/CPE.

Furthermore, the reproducibility of the presented sensing platform for L-DOPA determination was examined, as well as the stability of the developed sensor. From five successive measurements of a 50 μM L-DOPA standard solution, the reproducibility was investigated. The obtained current values were 2.15 µA, 2.26 µA, 2.20 µA, 2.18 µA and 2.00 µA, giving an RSD of 4.50%. The acquired results prove the remarkable sensitivity and reproducibility of the proposed sensor. The stability of the electrode was estimated by recording the DPV response of 50 μM of L-DOPA standard solution during a one-month period. During the mentioned period, the electrode was stored under laboratory conditions. Analyses were performed on the 1st, 3rd, 5th, 10th, and 30th days, counting from the day of sensor preparation. The relative standard deviation of these measurements was 6.12%, while the final current decrease was lower than 5.12% from the initial value. This study indicates that the proposed preparation procedure and developed sensor have good stability over the tested period.

The proposed sensor was compared with the recently reported ones to detect L-DOPA. The obtained results are comparable to already described sensors found in the literature of last few years, as seen in Table 1.

### 3.6. Interferences

**Figure 7:**
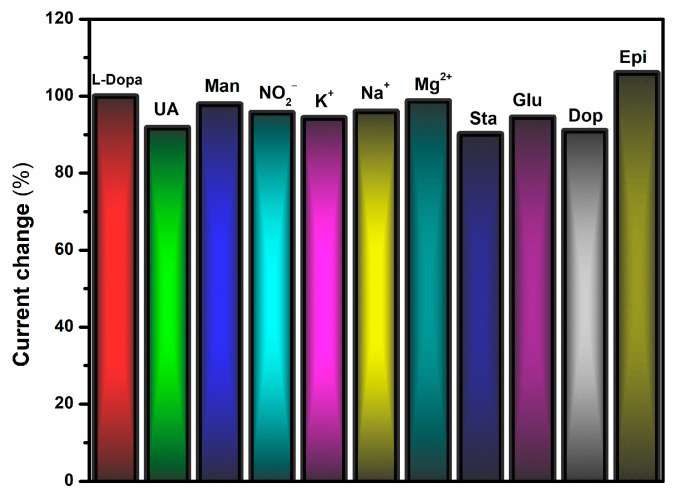
Interferences study.

When developing a new analytical method for detecting a specific analyte, it is necessary to examine the selectivity of the proposed sensor. Our research investigated organic compounds commonly present in L-DOPA tablets (mannitol (Man), starch (Sta), and glucose (Glu)) and agreeable metal ions (K^+^, Mg^2+^, Na^+^) as interfering species. Furthermore, we examined the influence of compounds potentially present in a biological matrix (uric acid (UA), dopamine (Dop), and epinephrine (Epi)) on sensor response toward L-DOPA. We added a certain amount (300 µL) of interference standard solutions (1 × 10^−4^ M) to the 30 mL L-DOPA solution (BRBS, pH 7) with a concentration of 1 × 10^−6^. This way, interference concentrations equivalent to the concentration of the L-dopa solution were achieved. Although it is almost impossible to find interfering substances in real samples at such a concentration, we wanted to show that even at high concentrations, the electrode is highly selective for the desired analyte. Figure 7 shows the change in the current intensity of the oxidation peak of L-DOPA after adding the tested interfering substance. Examined cations and anions, mannitol, glucose, and uric acid did not interfere with L-DOPA determination; the reductions in analyte current were less than 5%.

On the other hand, dopamine and epinephrine showed interference in the L-DOPA when they were in the solution in the same concentration as the analyte. Therefore, the selectivity of the proposed sensor is satisfactory regarding pharmaceutical tables as analytes. On the other hand, if this sensor is used for biological samples, it requires some pre-sample treatment steps to eliminate dopamine and epinephrine interference.

### 3.7. Real Sample

The practical applicability of the Eu_2_O_3_@Cr_2_O_3_/CPE sensor was tested by determining the L-DOPA concentration in real samples of “Madopar” tablets. Before the examination, “Madopar” tablet samples were pre-treated according to the method explained in the experimental section. The proposed analytical method for L-DOPA detection was validated using a recovery test using a prepared tablet solution for standard addition. Samples containing different amounts of “Madopar” solution were recorded, and the L-DOPA concentration was calculated based on the linear response of the proposed analytical procedure. Recovery values ranged from 95.7 to 98.6% (Table 2). Additionally, the presented method was used to determine the L-DOPA content in Madopar tablets, and the acquired results were compared with the declared values. The average concentration of L-DOPA in tablets obtained using the developed sensor was 98 ± 3 mg/tablet (100 mg/tablet). As can be seen, these results are very close to the values declared on the label. Excellent agreement of measured values with the labelled ones implies the manageable extension of the proposed procedure to various applications and feasible transfer to commercial application.

## 4. Conclusions

In this work, we integrated a hydrothermal synthesis procedure with the rare earth oxide to prepare a new nanohybrid material (Eu_2_O_3_@Cr_2_O_3_) for sensitive detection of L-DOPA, the important drug for Parkinson’s disease treatment. To the best of our knowledge, this was the first time this nanomaterial had been applied to electrochemical sensing. The morphological and electrochemical properties of the material were investigated using both spectroscopical and analytical methods. The prepared material was used to modify the carbon paste electrode, and the electrode functionalized in this way displayed improved electrocatalytic properties towards L-DOPA sensing. The large surface area and enhanced diffusion capacities resulted in a wide operating linear range of 1 to 100 µM, with the detection limit at a submicromolar range. The satisfactory selectivity, high stability, and excellent repeatability of the proposed method implies its application in real-time, world-wide sample analysis. The above-listed results proved that the developed electrocatalyst can serve as an efficient sensing probe for the monitoring of L-DOPA, with great potential for the technology transfer to an out-of-laboratory application.

## Figures and Tables

**Table 1:** Comparison of the recently reported literature for L-DOPA sensors.

Electrode	Method	Linear Range (µM)	LOD (µM)	Ref
Eu_2_O_3_@Cr_2_O_3_/CPE	DPV	1–100	0.72	This work
GC/CNT/PHQ/CE	DPV	0.005–20	0.000221	[5]
tyrosinase@ZIF-8/GO/Au	Amperometry	1–95	0.45	[36]
SWCNT-COOH@Nd_2_O_3_-SiO_2_	Amperometry	2–52	0.7	[37]
GP-CAc/PVC	SWV	8–100	0.06	[38]
pCoTAPc/SPCE	DPV	0.1–1000	0.03	[39]
Bi_2_Se_3_ NPs/rGO/Pt-E	DPV	6–250	0.23	[40]
AuNP/PPy/GCE	Amperometry	0.1–6	0.075	[41]
CuNPs/MWCNT-MIP	CV	0.01–1	0.009	[42]
Cysteic acid/GCE	DPV	0.35–4	0.11	[43]
RGO/TMU-22PE	SWV	0.1–85	0.02	[44]
MnO2@ IJPCNT	Amperometry	0.1–10	0.054	[45]
GO-Y_2_O_3_/GCE	SWV	0.5–350	0.05	[46]

Abbreviations: CPE—carbon paste electrode; ZIF-8—Zeolitic imidazolate framework; GO—graphene oxide; SWCNT—single wall carbon nanotubes; CAc—cellulose acetate; GP—graphite powder; PVC—polyvinyl chloride sheet; pCoTAPc—polytetraaminocobalt (II) phthalocyanine; SPCE—Screen printed carbon electrode; NP—nanoparticles; rGo—reduced graphene oxide; Pt-E—platinum electrode; PPY—polypyrrole; GCE—glassy carbon electrode; CE—Benzo-12-crown-4; PHQ—Poly-hydroquinone; CNT—carbon nanotubes; MWCNT—multiwalled carbon nanotubes; MIP—molecular imprinted polymer; IJPCNT—inkjet-printed carbon nanotube electrode.

**Table 2:** Determination of L-Dopa in the “Madopar” tablet sample at Eu_2_O_3_@Cr_2_O_3_ modified CPE.

Added (µM)	Found (µM)	Recovery (%)
5	4.96	98.6
10	9.73	97.3
20	19.24	96.2
40	38.28	95.7

## Data Availability

Not applicable.
